# Supplementary material for: Multi‐dataset identification of innovative feature genes and molecular mechanisms in keratoconus
Source: J Cell Mol Med. 2024 Sep 19;28(18):e70079. doi: 10.1111/jcmm.70079 (PMC11412914; doi:10.1111/jcmm.70079)
Supplement: Supplementary file 2 — Material S2. [file JCMM-28-e70079-s002.zip › 7.GSEA analysis/4.MYRF/KEGG_FOCAL_ADHESION.html]

Details for gene set KEGG\_FOCAL\_ADHESION[GSEA]

|  || Dataset | MYRF.group.cls#h\_versus\_l |
| Phenotype | group.cls#h\_versus\_l |
| Upregulated in class | l |
| GeneSet | KEGG\_FOCAL\_ADHESION |
| Enrichment Score (ES) | -0.4131204 |
| Normalized Enrichment Score (NES) | -1.4453282 |
| Nominal p-value | 0.08401639 |
| FDR q-value | 0.21182345 |
| FWER p-Value | 0.829 |
Table: GSEA Results Summary

  

Fig 1: Enrichment plot: KEGG\_FOCAL\_ADHESION      
 Profile of the Running ES Score & Positions of GeneSet Members on the Rank Ordered List

  

| SYMBOL | TITLE | RANK IN GENE LIST | RANK METRIC SCORE | RUNNING ES | CORE ENRICHMENT || 1 | COL6A6 | na | 599 | 0.547 | 0.0006 | No |
| 2 | PIK3R5 | na | 661 | 0.528 | 0.0162 | No |
| 3 | PDGFRA | na | 1428 | 0.407 | 0.0074 | No |
| 4 | ITGA4 | na | 1618 | 0.389 | 0.0147 | No |
| 5 | RAC3 | na | 2468 | 0.325 | 0.0008 | No |
| 6 | ELK1 | na | 3767 | 0.262 | -0.0282 | No |
| 7 | LAMB4 | na | 3997 | 0.254 | -0.0265 | No |
| 8 | VWF | na | 5297 | 0.213 | -0.0571 | No |
| 9 | TNR | na | 5527 | 0.207 | -0.0569 | No |
| 10 | ITGA9 | na | 5848 | 0.197 | -0.0597 | No |
| 11 | SOS1 | na | 6706 | 0.172 | -0.0789 | No |
| 12 | COL1A2 | na | 6809 | 0.170 | -0.0763 | No |
| 13 | FN1 | na | 6837 | 0.168 | -0.0715 | No |
| 14 | TNXB | na | 6939 | 0.166 | -0.0690 | No |
| 15 | COL1A1 | na | 6983 | 0.164 | -0.0649 | No |
| 16 | PIK3R1 | na | 7105 | 0.161 | -0.0631 | No |
| 17 | IGF1R | na | 7135 | 0.160 | -0.0587 | No |
| 18 | BRAF | na | 8198 | 0.133 | -0.0850 | No |
| 19 | COL4A1 | na | 8213 | 0.133 | -0.0811 | No |
| 20 | MAPK9 | na | 8409 | 0.129 | -0.0825 | No |
| 21 | IGF1 | na | 8743 | 0.121 | -0.0881 | No |
| 22 | ROCK1 | na | 8751 | 0.121 | -0.0843 | No |
| 23 | ITGB7 | na | 9321 | 0.109 | -0.0972 | No |
| 24 | IBSP | na | 9361 | 0.108 | -0.0948 | No |
| 25 | RASGRF1 | na | 9396 | 0.108 | -0.0922 | No |
| 26 | BCAR1 | na | 9571 | 0.104 | -0.0939 | No |
| 27 | PIK3CG | na | 9794 | 0.100 | -0.0970 | No |
| 28 | COL11A1 | na | 9811 | 0.099 | -0.0942 | No |
| 29 | FLT4 | na | 9822 | 0.099 | -0.0913 | No |
| 30 | AKT2 | na | 10210 | 0.092 | -0.0994 | No |
| 31 | FLNB | na | 10346 | 0.090 | -0.1004 | No |
| 32 | MYL5 | na | 10477 | 0.087 | -0.1013 | No |
| 33 | PDPK1 | na | 11113 | 0.076 | -0.1172 | No |
| 34 | PTEN | na | 11228 | 0.075 | -0.1180 | No |
| 35 | PIK3CA | na | 11255 | 0.074 | -0.1164 | No |
| 36 | LAMA5 | na | 11263 | 0.074 | -0.1141 | No |
| 37 | SHC3 | na | 11433 | 0.071 | -0.1167 | No |
| 38 | VAV2 | na | 11454 | 0.071 | -0.1149 | No |
| 39 | VAV3 | na | 11728 | 0.067 | -0.1206 | No |
| 40 | XIAP | na | 11781 | 0.066 | -0.1199 | No |
| 41 | LAMC3 | na | 12089 | 0.062 | -0.1268 | No |
| 42 | SPP1 | na | 12214 | 0.060 | -0.1284 | No |
| 43 | ITGB6 | na | 12239 | 0.060 | -0.1271 | No |
| 44 | MAPK10 | na | 12440 | 0.057 | -0.1310 | No |
| 45 | ACTB | na | 12485 | 0.057 | -0.1304 | No |
| 46 | PIK3R2 | na | 12535 | 0.056 | -0.1300 | No |
| 47 | SOS2 | na | 12783 | 0.053 | -0.1354 | No |
| 48 | ERBB2 | na | 13228 | 0.047 | -0.1467 | No |
| 49 | ARHGAP35 | na | 13305 | 0.046 | -0.1474 | No |
| 50 | ROCK2 | na | 13790 | 0.040 | -0.1601 | No |
| 51 | PDGFA | na | 13993 | 0.038 | -0.1647 | No |
| 52 | PRKCA | na | 14202 | 0.036 | -0.1696 | No |
| 53 | MYLK | na | 14280 | 0.035 | -0.1707 | No |
| 54 | MAPK3 | na | 14382 | 0.034 | -0.1725 | No |
| 55 | CCND1 | na | 14657 | 0.031 | -0.1794 | No |
| 56 | DIAPH1 | na | 14672 | 0.031 | -0.1788 | No |
| 57 | ACTN4 | na | 14881 | 0.029 | -0.1839 | No |
| 58 | CTNNB1 | na | 15600 | 0.022 | -0.2039 | No |
| 59 | MYL12B | na | 15972 | 0.018 | -0.2141 | No |
| 60 | PRKCG | na | 15973 | 0.018 | -0.2135 | No |
| 61 | JUN | na | 16671 | 0.011 | -0.2333 | No |
| 62 | FLT1 | na | 16677 | 0.011 | -0.2331 | No |
| 63 | EGFR | na | 17031 | 0.008 | -0.2430 | No |
| 64 | PIK3CB | na | 17199 | 0.007 | -0.2477 | No |
| 65 | RAC2 | na | 17259 | 0.006 | -0.2492 | No |
| 66 | CAPN2 | na | 17286 | 0.006 | -0.2497 | No |
| 67 | RAP1A | na | 17348 | 0.006 | -0.2513 | No |
| 68 | COL3A1 | na | 17558 | 0.004 | -0.2572 | No |
| 69 | PAK4 | na | 17893 | 0.001 | -0.2669 | No |
| 70 | COL5A3 | na | 18007 | -0.001 | -0.2701 | No |
| 71 | MYL12A | na | 18052 | -0.001 | -0.2714 | No |
| 72 | AKT1 | na | 18101 | -0.001 | -0.2727 | No |
| 73 | PPP1CB | na | 18335 | -0.004 | -0.2793 | No |
| 74 | PPP1CA | na | 18340 | -0.004 | -0.2793 | No |
| 75 | BIRC2 | na | 18537 | -0.006 | -0.2848 | No |
| 76 | MAPK8 | na | 18612 | -0.006 | -0.2868 | No |
| 77 | RHOA | na | 18827 | -0.008 | -0.2927 | No |
| 78 | RAP1B | na | 18871 | -0.009 | -0.2936 | No |
| 79 | EGF | na | 19142 | -0.012 | -0.3010 | No |
| 80 | ITGB4 | na | 19208 | -0.013 | -0.3025 | No |
| 81 | PAK3 | na | 19225 | -0.013 | -0.3025 | No |
| 82 | VASP | na | 19325 | -0.014 | -0.3050 | No |
| 83 | GSK3B | na | 19466 | -0.016 | -0.3085 | No |
| 84 | VEGFB | na | 20319 | -0.026 | -0.3323 | No |
| 85 | MAPK1 | na | 20406 | -0.027 | -0.3339 | No |
| 86 | PIK3CD | na | 20433 | -0.027 | -0.3338 | No |
| 87 | PIK3R3 | na | 20464 | -0.027 | -0.3338 | No |
| 88 | DOCK1 | na | 20532 | -0.028 | -0.3348 | No |
| 89 | COL6A3 | na | 20620 | -0.029 | -0.3364 | No |
| 90 | ARHGAP5 | na | 20650 | -0.029 | -0.3363 | No |
| 91 | PIP5K1C | na | 21353 | -0.038 | -0.3553 | No |
| 92 | PPP1R12A | na | 21522 | -0.040 | -0.3589 | No |
| 93 | RAF1 | na | 21720 | -0.042 | -0.3632 | No |
| 94 | PAK6 | na | 21811 | -0.043 | -0.3644 | No |
| 95 | HRAS | na | 21856 | -0.044 | -0.3642 | No |
| 96 | RAC1 | na | 21936 | -0.045 | -0.3650 | No |
| 97 | LAMB2 | na | 21964 | -0.045 | -0.3643 | No |
| 98 | RELN | na | 21970 | -0.045 | -0.3630 | No |
| 99 | THBS1 | na | 22189 | -0.048 | -0.3677 | No |
| 100 | ILK | na | 22215 | -0.048 | -0.3668 | No |
| 101 | ACTG1 | na | 22317 | -0.050 | -0.3681 | No |
| 102 | CHAD | na | 22755 | -0.056 | -0.3790 | No |
| 103 | COL11A2 | na | 22833 | -0.057 | -0.3793 | No |
| 104 | PPP1CC | na | 22860 | -0.057 | -0.3782 | No |
| 105 | LAMA2 | na | 23159 | -0.062 | -0.3848 | No |
| 106 | CDC42 | na | 23297 | -0.064 | -0.3866 | No |
| 107 | CCND3 | na | 23579 | -0.069 | -0.3925 | No |
| 108 | TLN2 | na | 23630 | -0.070 | -0.3917 | No |
| 109 | PTK2 | na | 23650 | -0.070 | -0.3899 | No |
| 110 | ITGA3 | na | 23712 | -0.071 | -0.3894 | No |
| 111 | ITGB5 | na | 23730 | -0.071 | -0.3875 | No |
| 112 | BAD | na | 23795 | -0.072 | -0.3870 | No |
| 113 | VCL | na | 24000 | -0.076 | -0.3904 | No |
| 114 | THBS3 | na | 24014 | -0.076 | -0.3883 | No |
| 115 | LAMB1 | na | 24119 | -0.078 | -0.3887 | No |
| 116 | GRB2 | na | 24215 | -0.079 | -0.3889 | No |
| 117 | SHC1 | na | 24263 | -0.080 | -0.3876 | No |
| 118 | BIRC3 | na | 24271 | -0.080 | -0.3852 | No |
| 119 | PAK2 | na | 24412 | -0.082 | -0.3865 | No |
| 120 | VTN | na | 25023 | -0.093 | -0.4011 | No |
| 121 | HGF | na | 25029 | -0.093 | -0.3982 | No |
| 122 | ITGA2 | na | 25150 | -0.096 | -0.3985 | No |
| 123 | CRK | na | 25398 | -0.101 | -0.4024 | No |
| 124 | PRKCB | na | 25770 | -0.108 | -0.4096 | Yes |
| 125 | CCND2 | na | 25792 | -0.109 | -0.4066 | Yes |
| 126 | TLN1 | na | 25864 | -0.110 | -0.4051 | Yes |
| 127 | ITGA2B | na | 25913 | -0.111 | -0.4028 | Yes |
| 128 | COL6A1 | na | 26040 | -0.114 | -0.4027 | Yes |
| 129 | COL4A2 | na | 26047 | -0.114 | -0.3992 | Yes |
| 130 | THBS4 | na | 26080 | -0.115 | -0.3963 | Yes |
| 131 | LAMA4 | na | 26157 | -0.116 | -0.3947 | Yes |
| 132 | PAK1 | na | 26325 | -0.120 | -0.3956 | Yes |
| 133 | PDGFB | na | 26504 | -0.123 | -0.3967 | Yes |
| 134 | THBS2 | na | 26821 | -0.131 | -0.4016 | Yes |
| 135 | ITGAV | na | 26862 | -0.132 | -0.3984 | Yes |
| 136 | ZYX | na | 26894 | -0.133 | -0.3949 | Yes |
| 137 | PDGFRB | na | 27235 | -0.141 | -0.4002 | Yes |
| 138 | FLNC | na | 27638 | -0.151 | -0.4068 | Yes |
| 139 | PARVG | na | 27703 | -0.153 | -0.4037 | Yes |
| 140 | ACTN3 | na | 27890 | -0.157 | -0.4039 | Yes |
| 141 | ACTN1 | na | 27985 | -0.160 | -0.4014 | Yes |
| 142 | LAMC1 | na | 27992 | -0.160 | -0.3963 | Yes |
| 143 | LAMC2 | na | 28053 | -0.162 | -0.3927 | Yes |
| 144 | ITGA11 | na | 28180 | -0.166 | -0.3909 | Yes |
| 145 | MYLK3 | na | 28223 | -0.167 | -0.3866 | Yes |
| 146 | PXN | na | 28410 | -0.173 | -0.3863 | Yes |
| 147 | RAPGEF1 | na | 28454 | -0.174 | -0.3819 | Yes |
| 148 | MYL9 | na | 28537 | -0.176 | -0.3785 | Yes |
| 149 | MYLK2 | na | 28598 | -0.178 | -0.3744 | Yes |
| 150 | VAV1 | na | 28682 | -0.181 | -0.3708 | Yes |
| 151 | MET | na | 28755 | -0.183 | -0.3669 | Yes |
| 152 | TNN | na | 29081 | -0.194 | -0.3699 | Yes |
| 153 | CRKL | na | 29092 | -0.194 | -0.3638 | Yes |
| 154 | ITGA7 | na | 29295 | -0.200 | -0.3631 | Yes |
| 155 | ITGA8 | na | 29346 | -0.201 | -0.3580 | Yes |
| 156 | LAMA3 | na | 29367 | -0.202 | -0.3519 | Yes |
| 157 | ITGA6 | na | 29414 | -0.203 | -0.3466 | Yes |
| 158 | ITGB3 | na | 29529 | -0.207 | -0.3431 | Yes |
| 159 | COL4A4 | na | 29690 | -0.213 | -0.3408 | Yes |
| 160 | ITGB1 | na | 29873 | -0.219 | -0.3388 | Yes |
| 161 | VEGFA | na | 29965 | -0.222 | -0.3342 | Yes |
| 162 | PARVA | na | 29981 | -0.223 | -0.3273 | Yes |
| 163 | COL2A1 | na | 30062 | -0.225 | -0.3222 | Yes |
| 164 | ACTN2 | na | 30149 | -0.229 | -0.3172 | Yes |
| 165 | MAP2K1 | na | 30150 | -0.229 | -0.3096 | Yes |
| 166 | LAMB3 | na | 30260 | -0.233 | -0.3052 | Yes |
| 167 | MYL2 | na | 30601 | -0.246 | -0.3069 | Yes |
| 168 | FLNA | na | 30801 | -0.254 | -0.3043 | Yes |
| 169 | ITGB8 | na | 30931 | -0.260 | -0.2995 | Yes |
| 170 | SRC | na | 31183 | -0.273 | -0.2978 | Yes |
| 171 | PGF | na | 31338 | -0.282 | -0.2930 | Yes |
| 172 | COL5A2 | na | 31481 | -0.290 | -0.2876 | Yes |
| 173 | COL6A2 | na | 31492 | -0.290 | -0.2784 | Yes |
| 174 | MYL7 | na | 31601 | -0.295 | -0.2718 | Yes |
| 175 | COL5A1 | na | 31692 | -0.301 | -0.2645 | Yes |
| 176 | ITGA10 | na | 32096 | -0.323 | -0.2656 | Yes |
| 177 | SHC4 | na | 32535 | -0.353 | -0.2667 | Yes |
| 178 | PDGFD | na | 32544 | -0.354 | -0.2553 | Yes |
| 179 | COMP | na | 32809 | -0.373 | -0.2507 | Yes |
| 180 | BCL2 | na | 32817 | -0.374 | -0.2386 | Yes |
| 181 | COL4A6 | na | 33147 | -0.402 | -0.2350 | Yes |
| 182 | MYL10 | na | 33204 | -0.407 | -0.2232 | Yes |
| 183 | TNC | na | 33271 | -0.416 | -0.2115 | Yes |
| 184 | VEGFC | na | 33298 | -0.419 | -0.1985 | Yes |
| 185 | KDR | na | 33306 | -0.420 | -0.1849 | Yes |
| 186 | PARVB | na | 33556 | -0.451 | -0.1773 | Yes |
| 187 | CAV1 | na | 33722 | -0.474 | -0.1665 | Yes |
| 188 | ITGA1 | na | 33768 | -0.482 | -0.1520 | Yes |
| 189 | FYN | na | 33971 | -0.517 | -0.1409 | Yes |
| 190 | CAV2 | na | 34014 | -0.526 | -0.1248 | Yes |
| 191 | LAMA1 | na | 34028 | -0.528 | -0.1078 | Yes |
| 192 | ITGA5 | na | 34505 | -0.679 | -0.0993 | Yes |
| 193 | CAV3 | na | 34583 | -0.727 | -0.0777 | Yes |
| 194 | PDGFC | na | 34611 | -0.758 | -0.0536 | Yes |
| 195 | SHC2 | na | 34640 | -0.800 | -0.0281 | Yes |
| 196 | AKT3 | na | 34696 | -0.932 | 0.0009 | Yes |
Table: GSEA details [plain text format]

  

Fig 2: KEGG\_FOCAL\_ADHESION      
 Blue-Pink O' Gram in the Space of the Analyzed GeneSet

  

Fig 3: KEGG\_FOCAL\_ADHESION: Random ES distribution      
 Gene set null distribution of ES for **KEGG\_FOCAL\_ADHESION**
